# PP36 Comparing Artificial-Intelligence-Generated And Expert-Driven Chemotherapy Recommendations For Node-Positive Breast Cancer

**DOI:** 10.1017/S0266462325102559

**Published:** 2025-12-29

**Authors:** Carlos Alberto Magliano, Bruno Barros

## Abstract

**Introduction:**

Generative artificial intelligence (AI) is transforming the generation of real-world evidence in health care. This study compares chemotherapy recommendations for women with node-positive breast cancer, as provided by a Delphi panel of experts, with those generated by ChatGPT and Copilot. The objective was to assess concordance and discrepancies between AI-generated and expert-driven recommendations.

**Methods:**

A Delphi panel of 10 independent breast cancer experts, blinded to each other’s responses, participated in an online survey to evaluate chemotherapy recommendations for early stage, node-positive breast cancer, stratified by menopausal status and Oncotype DX Breast Recurrence Score® (RS: <14, 14 to 25, >25). ChatGPT and Copilot, using automatic prompt engineering, addressed the same scenarios as agent-based oncologists. Expert recommendations were summarized as arithmetic means, while AI responses were analyzed for concordance. A one-sample t-test compared mean estimates between the Delphi panel and each AI tool.

**Results:**

Chemotherapy recommendation rates for premenopausal women by the Delphi panel, ChatGPT, and Copilot were: RS less than 14 (82.2, 2.5, 10); RS 14 to 25 (96.7, 22.5, 30); RS greater than 25 (100, 80, 60). For postmenopausal women, these were: RS less than 14 (3.3, 1.5, 15); RS 14 to 25 (17.8, 17.5, 35); RS greater than 25 (94.4, 72.5, 65). Significant differences were observed, especially in lower RS ranges. Differences in means between the Delphi panel and AI for postmenopausal with a RS greater than 25 and premenopausal with a RS less than or equal to 25 were statistically significant (p<0.05).

**Conclusions:**

The recommendations from AI demonstrated significant divergence from the “gold standard” panel, especially for premenopausal patients. The observed deviations from Brazilian experts’ recommendations likely stem from differences in local practices, compared with international published data, which may have influenced AI outputs. These findings emphasize the continued importance of human panels to account for regional variations in clinical decision-making.

